# Hospitals and the Problem of Reform

**Published:** 1911-06-24

**Authors:** 


### HOSPITALS AND THE PROBLEM OF REFORM.

We have for some time past been pointing out in these columns the splendid opportunity that recent developments have offered to hospital workers to reform and amend our hospital system in details which the experience of the last quarter of a century has shown are capable of improvement and alteration for the better. The issue of the reports by the Commissioners on the Poor Law was one of these developments; the publication of the Government scheme of compulsory invalidity insurance seems another which is equally important and equally likely to have far-reaching effects on the voluntary institutions of this country. It is therefore of the greatest importance that hospital workers should be adequately and accurately informed with regard to the possibilities before them. We need statistical and general information on many points which are at present vague before we can be in a position to state definitely along what lines progress should be attempted. For instance, there is at the present moment a good deal of loose talk about the effect which the introduction of compulsory invalidity insurance has had upon the hospitals in other countries. The vague phrase "other countries," which politicians and the lay Press are so fond of using, means in this connection only Germany and Austria-Hungary; no other country has invalidity insurance systems which are likely to affect their hospitals to any extent, since insurance in Italy, France, and Poland has hitherto had no effect upon the revenue or work of the hospitals in these countries, while it is probable that the adoption of the system by Switzerland will be entirely upon German lines and that the results in the Confederation, so far as the hospitals are concerned, will be similar to those which have been recognised in Germany since 1889. That being the case, a consideration of the hospital system in Germany and Austria and the effect which the insurance laws have had upon that system, becomes of the utmost importance at this juncture if we wish to benefit by the lessons that administrators have learned from the insurance laws and their effect generally. We have therefore been at some pains, since the hospital question became increasingly urgent, to collect all the available data, which we are now publishing in a series of special articles which deal fully with the whole question of hospitals in Germany, and show in what manner and to what extent their work has been influenced by insurance. The conclusions to be drawn from a study of the facts are evident to everyone who takes the trouble to glance over the articles. We would wish, however, to lay special stress on one or two of these points. One is the fact that the introduction of social legislation of the character of the insurance Acts has tended to promote co-operation between the State and the hospitals to an extent which is unknown in any other European country; another is the fact that it has materially raised the status of all hospitals in the Empire, and has led to the adoption of a system of pay wards, the results of which have been a noteworthy increase in the hospital revenues.

These two points are worth consideration by every hospital worker in this country. The Hospital has strongly supported the adoption of some modification of our present system by which we can acquire the same benefits which the frank acceptation of the rule that the State, as such, and the community at large, possess a material interest in the welfare of hospitals, has brought to the German institutions. Similarly, we have urged for many years the adoption of a system of pay wards. Readers of The Hospital need not be reminded that our policy in the past has been to support, as much as lay in our power to do so, the proposals that patients should be asked to pay for the food, medicines, bandages, and other things supplied to them, and we have pointed out that if some such system were adopted it would go far to help the progress of the hospitals. While we have condemned State control as likely to cripple hospital progress and destroy efficiency in the highest and best meaning of the term, we have always insisted on the obligations of the community to support the hospitals, and while we have pointed out that the London hospitals are in a very good financial position, we are quite aware that the introduction of an extensive system of State-aided insurance, or of wide-reaching modifications of our Poor Law, are likely to interfere seriously with the work of the hospitals, and that it is therefore necessary to call for some *quid pro quo* from the community that does not, as a section, support the hospitals. It is of the very greatest importance that the nature of such a *quid pro quo* to be offered to the hospitals should be carefully studied. We do not agree with the Chancellor of the Exchequer that it is desirable to chop and change about every few years. Instability in a system of such national importance as that of the hospitals breeds distrust and unpopularity, and it is highly desirable that we should frame a line of policy and apply it, after serious and thoughtful consideration, without shrinking from the inevitable consequences that it may bring about. The principle of co-operation has already been recognised in a few of our public institutions, such as the Manchester Infirmary. If it can be extended so as to include all our voluntary hospitals—and we believe that it can be so extended without necessarily bringing into being a system of State supervision and State control—it would be in the interests of both the community and the institutions. The details of the scheme demand careful working out. We hope in a future issue to deal with this side of the matter at some length. Meanwhile we commend to our readers a study of the conditions under which co-operation exists in other parts of the world and of the effects which the introduction of insurance schemes have had upon the work and policy of the hospitals elsewhere. Such a study will enable them to have in mind a clear idea of the leading points which should be remembered in a discussion of this subject, to discriminate between the loose and didactic statements of those who are obviously unacquainted with the facts, and to draw certain conclusions. These conclusions, we venture to think, will tend to reveal that there is a possibility of remodelling our hospital system along lines which will be favourable to the development and extension of the best that we now possess and to the acquisition and acclimatisation of the best that is to be found in other systems of hospital relief.

